# Q&A: How do plants sense and respond to UV-B radiation?

**DOI:** 10.1186/s12915-015-0156-y

**Published:** 2015-06-30

**Authors:** Roman Ulm, Gareth I Jenkins

**Affiliations:** Department of Botany and Plant Biology, University of Geneva, Sciences III, CH-1211, Geneva, 4 Switzerland; Institute of Molecular, Cell and Systems Biology, College of Medical, Veterinary and Life Sciences, University of Glasgow, Glasgow, G12 8QQ UK

## Abstract

Plants are able to sense UV-B through the UV-B photoreceptor UVR8. UV-B photon absorption by a UVR8 homodimer leads to UVR8 monomerization and interaction with the downstream signaling factor COP1. This then initiates changes in gene expression, which lead to several metabolic and morphological alterations. A major response is the activation of mechanisms associated with UV-B acclimation and UV-B tolerance, including biosynthesis of sunscreen metabolites, antioxidants and DNA repair enzymes. To balance the response, UVR8 is inactivated by regulated re-dimerization. Apart from their importance for plants, UVR8 and its interacting protein COP1 have already proved useful for the optogenetic toolkit used to engineer synthetic light-dependent responses.

## What is UV-B radiation?

Ultraviolet (UV) radiation is the portion of the electromagnetic spectrum between X-rays and visible light that is conventionally divided into UV-A (315 to 400 nm), UV-B (280 to 315 nm) and UV-C (100 to 280 nm) radiation. Whereas UV-C is entirely absorbed by the stratospheric ozone layer, UV-A and some UV-B radiation reach the Earth’s surface and thus can affect the biosphere. UV-B is the most harmful form of radiation from sunlight reaching the Earth mainly because it can cause photochemical DNA damage. Exposure to UV-B is thus particularly notorious for being a major risk factor for most human skin cancers.

## Why is UV-B an issue for plants?

DNA is chemically the same in all organisms and thus similarly susceptible to damage by UV-B in plants and humans. Things are, however, additionally complicated for plants, as they cannot escape UV-B by behavioral responses (for example, moving to shadow, putting on clothes or sun cream). Rather, plants are sessile organisms that depend on sunlight as the energy source for photosynthesis. Thus, they need to achieve a balance between optimal light capture and UV-B protection. One way to escape UV-B exposure is by limiting their life to times/places when/where UV-B levels are lower (such as spring, autumn, in shade under canopy). However, several plants, including crops, grow during summer under conditions with high UV-B without being sunburned, which can be appreciated in a green landscape on a hike in summer. But growth in bright sunlight is hazardous - simply consider a human exposed to sunlight during the entire day without clothes and sun cream protection! Thus, efficient UV-B protection mechanisms are of great importance to plants.

## Can plants specifically ‘see’ UV-B?

**Fig. 1:**
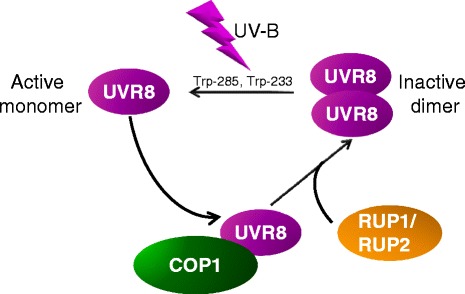
The UVR8 photocycle. UV-B photon absorption by the UVR8 homodimer via a tryptophan-based mechanism results in UVR8 monomerization. The UVR8 monomer interacts directly with COP1 to initiate UV-B signaling, which results in changes in gene expression (including the induction of *RUP1* and *RUP2* expression) and ultimately regulates UV-B-induced photomorphogenesis and stress acclimation. Re-dimerization of UVR8 through the action of RUP1 and RUP2 disrupts the UVR8-COP1 interaction and regenerates the UVR8 homodimer again ready for UV-B perception. Image reprinted from [46]: thearabidopsisbook.org Copyright American Society of Plant Biologists

Yes, plants can directly sense UV-B photons through a specific UV-B photoreceptor called UV RESISTANCE LOCUS 8 (UVR8) [1]. UVR8 is a seven-bladed β-propeller protein that exists as a homodimer held together by interactions between charged amino acids [1–3]. After UV-B absorption the UVR8 homodimer converts to monomers that initiate a signaling cascade, which ultimately leads to transcriptional regulation of target genes [1, 4, 5] (Fig. 1). The amount of UVR8 monomer is a measure for the prevailing UV-B levels, allowing appropriate responses. Importantly, UVR8 induces UV-B acclimation and UV-B tolerance [4–6]. Acclimation involves, for example, increased levels of DNA repair enzymes and accumulation of ‘sunscreen’ metabolites that absorb UV-B and thereby protect underlying tissues.

## How exactly does UVR8 act as a photoreceptor?

**Fig. 2:**
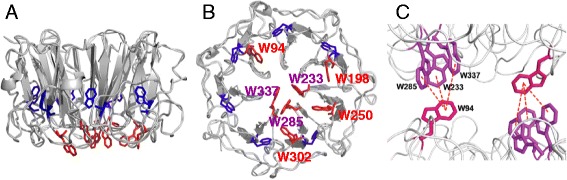
UVR8 structure and arrangement of its 14 tryptophans. **a**,**b** All UVR8 tryptophans in the monomer viewed from the side (except W400, which is in the carboxy-terminal domain that was not included in the solved structures, here shown for amino acids 14 to 380) (**a**) and from the dimeric interaction surface (**b**). Tryptophans in the protein core (blue) and at the interaction surface (red) are distinguished. Images modified from [8]. **c** Pyramidal arrangement of tryptophans across the UVR8 dimer interface. The triad tryptophans W233, W285 and W337 on one monomer of UVR8 are closely associated with W94 on the opposing monomer. Image modified from [7]

UVR8 is unique among photoreceptors in that it does not employ a bound chromophore to absorb light of a particular spectral quality [2, 3]. Instead UVR8 uses specific tryptophans in its primary sequence for UV-B photoreception [1–3, 7]. UVR8 has 14 tryptophans whose positions in the protein are highly conserved in evolution. Six of the tryptophans are in the β-propeller blades of the protein and appear to be important in maintaining blade structure; one tryptophan, of unknown function, is in the carboxy-terminal domain, and the remaining seven are arranged at the surface where two UVR8 monomers interact to form the dimer [7, 8] (Fig. 2a,b). Three of the tryptophans at the dimer interface (W233, W285 and W337; W = tryptophan in the amino acid single-letter code) are in very close proximity, and this ‘triad’ of tryptophans is close to W94 on the opposing monomer, forming a pyramidal arrangement. There are two such tryptophan ‘pyramids’ across the dimer (Fig. 2c).

Mutational studies, both *in vitro* with the purified protein [2, 3], and *in vivo* in yeast [1, 8] and plants [2, 8–10], implicate specific tryptophans at the dimer interface as being crucial for photoreception. In particular, mutation of either W285 or W233 to phenylalanine greatly impairs UVR8 function, indicating that these tryptophans have crucial, non-redundant roles in UVR8 photoreception. The function of the remaining tryptophans is poorly understood, although it has been suggested that tryptophans outside the pyramid absorb UV-B and transfer excitation to W233/W285 [11–13]. Furthermore, it is not clear how UV-B absorption by specific tryptophans actually leads to dissociation of the UVR8 dimer, which initiates signaling. Dimer integrity is maintained by electrostatic interactions between charged amino acids across the dimer interaction surface. Interactions between charged amino acids, notably R286 (R = arginine) with D96 and D107 (D = aspartic acid), and R338 with D44 and E43 (E = glutamic acid), are particularly important in maintaining the dimer, since mutations in these amino acids lead to weakening of the dimer or constitutive monomerization [2, 3]. The key charged amino acids are adjacent to the triad tryptophans, suggesting that UV-B excitation of tryptophans could result in proton-coupled electron transfer to specific charged amino acids, neutralizing interactions that maintain the dimer [2, 12, 14, 15]. In addition, there is evidence, from dynamic crystallography, that UV-B photoreception causes reorientation of W233 and W285, leading to ejection of an adjacent water molecule, which could weaken hydrogen bonds between monomers [13]. Associated conformational changes may further promote dimer dissociation.

Thus, it has been established that UVR8 has a novel, tryptophan-based mechanism of UV-B photoreception, but further details of the processes involved remain to be elucidated.

## How does UVR8 initiate signal transduction and transcriptional regulation?

UVR8 seems to be mainly active in the nucleus of plant cells. Despite being rather abundant in the cytoplasm as well, no cytoplasmic function of UVR8 has yet been reported. Indeed, UV-B activation results in the nuclear accumulation of UVR8 [16]. The UV-B-activated UVR8 monomer interacts with CONSTITUTIVELY PHOTOMORPHOGENIC 1 (COP1) [5] (Fig. 1), an E3 ubiquitin ligase [17]. COP1’s function as a central repressor of photomorphogenesis requires accessory proteins of the SPA family and is associated with its substrate receptor function of CUL4-DDB1-based E3 ubiquitin ligase complexes [17, 18]. COP1 thereby targets several positive regulators of light responses for proteasomal degradation, particularly in darkness [17]. In light this activity is reduced through the activity of photoreceptors sensing visible light. Thus, in addition to UVR8, the red/far-red and blue light photoreceptors (phytochromes and cryptochromes, respectively) impinge on the activity of COP1 [17]; but how COP1 integrates these different inputs remains unknown. Independent of this, phytochromes and cryptochromes interact light-dependently with the SPA proteins, which consequently inactivates COP1 [19, 20]. It is interesting to note that UVR8 is the only photoreceptor to show a light-dependent interaction directly with COP1, independent of COP1 accessory SPA proteins [1, 21]. Similarly, the COP1 activity for early UV-B responses is independent of the SPA proteins [22]. Thus, even though phytochromes, cryptochromes and UVR8 all regulate COP1 function, the underlying mechanisms are apparently different.

There is evidence that two separate domains of UVR8 mediate the interaction with COP1: the β-propeller core and the carboxy-terminal C27 domain. Monomerization of the UVR8 core domain is responsible for the UV-B-dependent interaction with COP1, which regulates COP1 activity through the C27 domain [23, 24]. Interaction of UVR8 with COP1 was found to dissociate the COP1-SPA core complex from CUL4-DDB1, forming a unique complex with UVR8 [18]. Interaction of UVR8 with COP1 stabilizes the ELONGATED HYPOCOTYL 5 (HY5) transcription factor [5, 18], enhances the association of HY5 with target promoters, including its own [25], and activates transcription of many UVR8-regulated genes [4, 22, 26, 27]. Many of these target genes are associated with UV-B protection and UV-B-damage repair [4–6, 26].

## How is UVR8 inactivated?

Signaling pathways usually include negative feedback regulation to balance their output. In the case of UVR8 signaling, an exaggerated response would result in impaired growth and dwarfism (as illustrated, for example, by experimental UVR8 overexpression) [5]. Moreover, in general, receptors need to be inactivated and revert to their ground state in the absence of a stimulus. Indeed, UVR8 monomers can revert to homodimers *in vivo* in the absence of UV-B to regenerate the active photoreceptor [28, 29]. This involves the action of the REPRESSOR OF UV-B PHOTOMORPHOGENESIS (RUP) 1 and 2 proteins that act as negative regulators of UV-B signaling by promoting the reversion of UVR8 to the homodimeric conformation [28, 30] (Fig. 1). This UVR8 re-dimerization activity requires direct interaction of RUP1 and RUP2 with the UVR8 C27 domain [23, 24, 29]. However, in contrast to COP1, RUP1 and RUP2 are also able to interact with C27 in the homodimeric form of UVR8 [23, 24, 30]. Interestingly, *RUP1* and *RUP2* gene expression is induced by UV-B through UVR8 signaling, providing a negative feedback loop to balance the UV-B response [30].

## Can UVR8 be found in all plants? And beyond?

Possible UVR8 orthologs can be identified in all plants where sequence information is available, including in single-celled green algae [1]. UVR8 signaling thus appears to be an evolutionarily ancient mechanism, consistent with a role in UV-protection of photosynthetic organisms [1]. UVR8 may indeed have been important early in the evolution of photosynthetic organisms to cope with the even higher doses of UV-B at that time and may have thus also helped the transition to terrestrial life, concomitant with the generation of the stratospheric ozone layer [31]. At present, there is, however, no evidence for the presence of a UVR8-type UV-B receptor outside of the plant kingdom.

## What is the link between UV-B acclimation and UV-B stress responses?

Acclimation to UV-B involves a combination of protective as well as repair measures, including the accumulation of UV-B-absorbing ‘sunscreen’ metabolites in the vacuoles of epidermal cells, increased levels of anti-oxidants, protection of the photosynthetic apparatus, and increased levels of DNA repair enzymes. As a result, as long as plants are well acclimated, they seem not to ‘feel’ UV-B as a stress that negatively and visibly interferes with cellular activities (also named distress) [32]. Plants can be considered UV-B-stressed when the UV-B levels and resulting cellular damage cannot be coped with (for example, [33]).

## Is UV-B acclimation the only response to UV-B mediated by the UV-B photoreceptor?

**Fig. 3:**
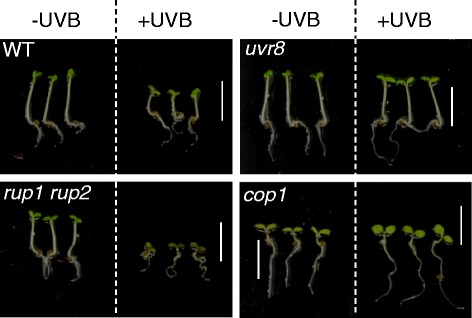
UV-B-induced hypocotyl growth inhibition in *Arabidopsis* seedlings. UV-B-mediated inhibition of hypocotyl elongation is apparent in four-day-old wild-type (WT) seedlings but not in *uvr8* or *cop1* mutants. This UV-B-induced photomorphogenic response is enhanced in *rup1 rup2* double mutant seedlings. Wild-type and mutant seedlings were grown under white light with or without supplementary narrowband UV-B (according to [5]). Scale = 5 mm. Image reprinted from [46]: thearabidopsisbook.org Copyright American Society of Plant Biologists

No, UVR8 seems to have a much broader effect on plant growth and development than only mediating UV-B acclimation. For example, UVR8 has also been implicated in UV-B-mediated entrainment of the circadian clock [34], hypocotyl growth inhibition [5] (Fig. 3), stomatal closure [35], phototropic bending [36], leaf development [37], inhibition of shade avoidance [38, 39], and osmotic stress [40] and pathogen responses [41]. However, some of these responses could be associated with UV-B stress acclimation, although this remains to be demonstrated.

## Could UVR8 potentially contribute to the optogenetic toolkit?

Optogenetics refers to the use of genetically encoded tools that allow the control of cellular activities by light [42]. Indeed, recently the first applications of UVR8 and the UVR8-COP1 interaction as an optogenetic tool were described [43–45]. UVR8 was used to engineer a UV-B-triggered protein secretion system where UVR8 fusion proteins were conditionally sequestered in the endoplasmic reticulum and UV-B allowed regulated trafficking to the plasma membrane [43]. The UVR8-COP1 interaction was used, for example, to develop UV-B-inducible expression systems in mammalian cells by split transcription factors that were rendered UV-B-responsive through fusion to UVR8 and COP1 [44, 45], similar to yeast two-hybrid assays [1]. Advantages of the UVR8 system include i) no application of an exogenous chromophore is needed, ii) experiments are possible in visible light (very specific and sensitive), and iii) there is no interference with imaging of the commonly used fluorescent proteins. Moreover, the rather low reversion kinetics can be an advantage or disadvantage dependent on the application. A disadvantage may be the high energy and potentially damaging aspect of UV-B radiation. However, UV-B levels needed are rather low (certainly compared to sunlight) and only short exposure times are often required. Moreover, future developments implementing two-photon laser excitation of tryptophans may help to further establish UVR8-derived optogenetic systems.

## Where can I find out more about plant UV-B signaling?

For interested readers we propose several recent review articles as further introductory reading on the topic [7, 21, 46–48].
